# Development of gambling disorder after bariatric surgery: a call for research

**DOI:** 10.3389/fpubh.2023.1206938

**Published:** 2023-06-22

**Authors:** Yassir Abdul Rahim, Fernando Fernandez-Aranda, Susana Jimenez-Murcia, Anders Håkansson

**Affiliations:** ^1^Helsingborg Hospital, Skåne Region, Helsingborg, Sweden; ^2^Malmö Addiction Center, Clinical Research Unit, Skåne Region, Malmö, Sweden; ^3^Faculty of Medicine, Department of Clinical Sciences Lund, Psychiatry, Lund, Sweden; ^4^CIBER Fisiopatología Obesidad y Nutrición (CIBERobn), Instituto de Salud Carlos III, Barcelona, Spain; ^5^Clinical Psychology Unit, University Hospital of Bellvitge, L’Hospitalet de Llobregat, Barcelona, Spain; ^6^Department of Clinical Sciences, School of Medicine and Health Sciences, University of Barcelona, L’Hospitalet de Llobregat, Barcelona, Spain; ^7^Psychoneurobiology of Eating and Addictive Behaviors Group, Neurosciences Program, Bellvitge Biomedical Research Institute (IDIBELL), L’Hospitalet de Llobregat, Barcelona, Spain

**Keywords:** gambling, gambling disorder, problem gambling, behavioral addiction, bariatric surgery

## Abstract

The relationship between bariatric surgery and alcohol use disorder (AUD) suggests that there may be a parallel connection between bariatric surgery and gambling disorder (GD), although this has never been researched before. Here, we describe observations suggesting that patients undergoing bariatric surgery may develop gambling disorders after surgery. Obese, older adults, and women may be at particular risk of developing GD because of their higher susceptibility to somatic comorbidities. We call for research addressing factors affecting the development of GD in patients undergoing bariatric surgery and how this could be prevented.

## Introduction

Bariatric surgery, such as gastric bypass surgery, is a standard and cost-effective treatment for morbid obesity. Extreme obesity is associated with health risks and cardiovascular disease. Adams et al. (1) demonstrated that Roux-en-Y gastric bypass (RYGB) induces significant and rapid weight loss as well as improvements in cardiovascular and metabolic risk factors. Patients undergoing RYGB surgery have greater remission rates and a lower incidence of diabetes, dyslipidemia, and hypertension than severely obese participants in the control group (1). In a meta-analysis, Azam et al. (2) found that the prevalence of alcohol use disorder (AUD) increased 2 years after gastric bypass surgery. According to Blackburn et al. (3), previous studies [e.g. (4–6),] suggested that changes in pharmacokinetics and metabolism induced by RYGB may be responsible for increased alcohol intake after bariatric surgery. Patients may become intoxicated faster and require more time to sober up after consuming less alcohol than they did prior to surgery, which may result in increased alcohol consumption and the development of AUD (3). In addition, Blackburn et al. (3) reported that several hypotheses have been proposed in studies (4, 6–8) to explain changes in the pharmacokinetics of alcohol and physiological causes which results in altered alcohol metabolism after gastric bypass surgery. The rapid emptying of the gastric pouch may facilitate faster absorption of alcohol into the jejunum. Additionally, the reduced volume of the stomach results in less amount of alcohol dehydrogenase, a substance that partially metabolizes alcohol. A study by Ostlund et al. (5) found that patients who had undergone gastric bypass surgery were significantly more likely to develop alcohol use disorders than those who had undergone a restrictive procedure. Interestingly, a study by Wee et al. (9) reported an increase in high-risk alcohol use after bariatric surgery. Initially, it was believed that the increased risk of AUD was due to “addiction transfer,” in which patients replace food consumption with alcohol consumption (10). However, this argument does not explain why AUD develops years after gastric bypass surgery and not immediately (2). On the other hand, studies have found that people with gambling disorders are more likely to have a history of other addictive behaviors, such as substance use disorders or compulsive shopping (11–14). Overall, more research is needed to fully understand the possible relationship between “addiction transfer” and gambling disorder and to develop effective treatments for individuals who may be at risk for developing multiple addictions. In contrast to alcohol use disorder, no studies describe the relationship between obese patients undergoing bariatric surgery and gambling disorders. GD may be a consequence of “addiction transfer” in obese patients undergoing bariatric surgery. Lemon et al. (15) reported possible links between GD and food addiction and a general increase in the risk of GD in cases of mental health problems. Furthermore, there is also a general risk of poor lifestyle habits in GD patients.

## Gambling disorder, bariatric surgery, and gender differences

A large national retrospective study on GD and cardiovascular and respiratory diseases was conducted based on National Patient Register data retrieved from the Swedish National Board of Health and Welfare; parallel and separately, the possible connection with bariatric surgery was examined from the same data, hence this commentary. The study was conducted following the Declaration of Helsinki’s ethical principles and approved by the regional ethical review board of Lund University (Ref: 2019-01559). The data retrieved included all patients over the age of 18 with a diagnosis of GD in Swedish specialized healthcare (ICD-10 code F63.0, also known as “pathological gambling”) at any time between 2005 and 2019. Two gender-and age matched control subjects were randomly selected from the general population using data from the Statistics Sweden population register for each patient with GD. The physician recorded the diagnosis as an ICD-code in the patient’s medical record. All ICD-codes from patients were automatically transferred to the system. These ICD-codes were then extracted from the National Patient Register based on inpatient and outpatient visits and used for research purposes. The primary variables of this study were measured based on these ICD-codes. Cardiovascular diseases included hypertension diseases (ICD-10 codes I10–I15), ischemic heart diseases (I20–I25), diseases within lung circulation (I26–I28), other forms of heart diseases (I30–I52), cerebrovascular diseases (I60–I69), arterial diseases (I70–I79), and venous diseases (I80–I89). Respiratory diseases included the ICD-codes J40–J47. Furthermore, diabetes and obesity were defined as ICD-10 codes E10–E14 and E66, respectively. Psychiatric comorbidities were also examined as a variable in the binary logistic regression and defined as substance use disorders (F10–F19), affective disorders (F30–F39), and anxiety disorders, phobias, PTSD, obsessive–compulsive disorders, and dissociative syndromes (F40–F49). Bariatric surgery was defined as ICD-10 codes JDF10, JDF11, JDF96, and JDF97. A pre-surgery diagnosis of GD was defined as a diagnosis of GD at any time before undergoing bariatric surgery. A post-surgery diagnosis of GD was defined as a diagnosis of GD at any time after undergoing bariatric surgery. All collected data were organized and analyzed in SPSS Statistics Version 28. A total of 10,766 patients were included, and 3,592 had GD. The GD group consisted of 2,791 men (77.70%) and 801 women (22.30%), with a median age of 35 and 40 years, respectively. Compared to controls, the prevalence of bariatric surgery was higher among patients with gambling disorders (1.89%, *n* = 68 vs. 0.65%, *n* = 47; *p* < 0.001). According to our findings, women with GD had a higher prevalence of bariatric surgery than men (5.24%, *n* = 42 vs. 0.93%, *n* = 26; *p* < 0.001). We also found that 63.24% of patients undergoing bariatric surgery in this case–control sample were diagnosed with GD post-surgery, while 36.76% had been diagnosed with GD pre-surgery. Another interesting finding was that 73.81% of women undergoing bariatric surgery developed GD post-surgery compared to only 46.15% of men (*p* < 0.05). Factors associated with bariatric surgery; age, gender, gambling disorder, diabetes, cardiovascular, respiratory, and psychiatric diseases, were analyzed using binary logistic regression to examine whether any of these factors could predict whether obese patients would undergo bariatric surgery or not. The logistic regression showed that only age was associated with bariatric surgery. The odds of having bariatric surgery increase by 2.00% per year of age (OR = 1.02, 95% CI [1.00–1.04]).

## Discussion

In summary, our findings indicate that patients who undergo bariatric surgery may be diagnosed with GD following surgery. In addition, women who undergo bariatric surgery are likely to develop GD after surgery. These results suggest the need to assess gambling problems prior to bariatric surgery as well as include psychoeducational sessions on the risk of developing substance or behavioral addictions after surgery, especially in those cases where morbid obesity is associated with an eating disorder (such as binge eating disorder or bulimia nervosa), food addiction, or problems such as emotional eating. Likely, in these profiles of patients, the risk would be greater. Dickhut et al. (16) and Mitchell et al. (17) found no increase in other addictive behaviors, such as GD, during the first year and first 3 years, respectively, after bariatric surgery. Our results suggest that there is an increase in GD post bariatric surgery. However, further research is needed to validate these findings and examine this relationship. Patients undergoing bariatric surgery should be given particular emphasis concerning the possible increased risk of developing GD post-surgery.

## Data availability statement

The raw data supporting the conclusions of this article will be made available by the authors, without undue reservation.

## Ethics statement

The studies involving human participants were reviewed and approved by Regional ethical review board of Lund University (Ref: 2019-01559). The patients/participants provided their written informed consent to participate in this study.

## Author contributions

YA, FF-A, SJ-M, and AH were all responsible for the overall design and research idea. YA wrote the first draft of the manuscript. All authors contributed to the article and approved the submitted version.

## Funding

This study was funded by the AH’s general research funding from the Swedish state-owned gambling operator (AB Svenska Spel) and the regional health care service organization (Region Skåne). In no way were these organizations involved in the study. The funders had no role in the design of the study; in the collection, analysis, or interpretation of data; in the writing of the manuscript, or in the decision to publish the results.

## Conflict of interest

AH is a professor at Lund University, and his position at this university is financially supported by AB Svenska Spel, the state-owned gambling operator in Sweden. Also, he has obtained research funding from the independent research council of Svenska Spel as well as from the corresponding research council of the state-owned Swedish alcohol monopoly, Systembolaget. FF-A and SJ-M have received consultancy honoraria from Novo Nordisk and FF-A editorial honoraria as EIC from Wiley.

The remaining author declares that the research was conducted in the absence of any commercial or financial relationships that could be construed as a potential conflict of interest.

## Publisher’s note

All claims expressed in this article are solely those of the authors and do not necessarily represent those of their affiliated organizations, or those of the publisher, the editors and the reviewers. Any product that may be evaluated in this article, or claim that may be made by its manufacturer, is not guaranteed or endorsed by the publisher.
